# Colour-assisted variation in elytral ICP-OES-based ionomics in an aposematic beetle

**DOI:** 10.1038/s41598-020-79329-4

**Published:** 2020-12-17

**Authors:** Grzegorz Orłowski, Przemysław Niedzielski, Jerzy Karg, Jędrzej Proch

**Affiliations:** 1 Institute for Agricultural and Forest Environment, Polish Academy of Sciences, Bukowska 19, 60-809 Poznań, Poland; 2 Department of Analytical Chemistry, Adam Mickiewicz University, Uniwersytetu Poznanskiego 8, 61-614 Poznań, Poland; 3 Department of Nature Conservation, Faculty of Biological Sciences, University of Zielona Góra, Prof. Z. Szafrana 1, 65-516 ZielonaGóra, Poland

**Keywords:** Environmental sciences, Environmental monitoring

## Abstract

Very little is known about how the elemental composition (ionome) of an insect cuticle varies as a result of different colouration. Using inductively-coupled plasma optical emission spectrometry (ICP-OES), we established ionomic profiles in microsamples of two adjacent regions of an insect cuticle with a contrasting colour pattern, namely, the black and orange regions of the elytra of the aposematic burying beetle *Nicrophorus vespillo*. The analysis revealed 53 elements (ranging in atomic weight from Na to Bi) occurring above the detection limit. The frequency of detectability of individual elements varied strongly, and only ten elements (Ba, Cu, Fe, K, Mg, Mn, P, Rb, Sb and Zn) were present in concentrations exceeding the detection limit in all the samples. The sum of concentrations of all elements in the orange regions of the elytra was 9% lower than in the black ones. The opposite distribution was displayed by the rare earth elements (REEs), the sum of which was 17% lower in the black elytral regions than in the orange ones. The concentrations of six elements were significantly higher in the black than in the orange regions: Al (by 97%), Cu (41%), Mn (14%), Na (46%), Se (97%) and W (47%). The concentrations of essential elements measured in both the black and orange regions exhibited very considerable variance: Ca (σ^2^ = 1834; 1882, respectively), K (145; 82) P (97; 76), Na (84; 53), Mg (24; 26) and Ba (9; 13). This, in part, could be attributed to individual differences, e.g. those resulting from the consumption of animal carcasses of different quality/chemical composition, but interference between elements and the consequent lowering of measurement quality are also possible. We highlight the fact that deeper insight into the basic relationship between insect colouration and variation in elemental composition requires micro-sampling of the homogeneous layers of an exoskeleton.

## Introduction

The huge variety of colouration in insects has always attracted attention. The majority of insect colour(s) and their resulting appearance are inseparably connected with the presence of pigmentsin their exoskeleton^1–4^. In particular, the melanin (a pigment capable of binding a variety of diatomic metals) that is concentrated in dark-coloured patches of the body integument or dead tissues like hair or feathers is considered to be a sink for toxic chemical elements^1,5,6^. Although the chemical basis of insect colouration has been widely studied, very little is known about how the elemental composition (ionome; sensu Baxter^7^) of insect cuticles varies with their different colouration^4,5,8^. This gap in our knowledge appears in part to be due to methodological difficulties, especially the determination of the ionome in microsamples.

Whole insects are widely used in ecotoxicological (biomonitoring) studies^9,10^. It should be borne in mind, however, that there is an apparent partitioning of certain metals between the exoskeleton and the rest of the body, primarily the gut and internal organs. In beetles, for instance, up to 29% of total Cd can bind with the exoskeleton^11^. Moreover, a recent investigation has shown that exoskeleton-internal elemental pools in beetles can be correlated, at least for certain essential (Mg, K, Na and Mn) and non-essential (As, Cd and Ni) elements, and that exoskeletal metals are related to habitat variability, albeit to a lesser degree than the internal pool of metals^12^. Hence, basic information on colour-assisted variation in the elemental composition of insect exoskeletons is essential for studies in many biological disciplines, particularly in ecotoxicological, biochemical or even evolutionary contexts (as postulated by McGraw^5^). The same applies to elements such as rare earths (REEs), transition metals, metalloids and non-metals, since there is very little information about their quantities in living organisms^13,14^. Certain metals (such as Zn and Mn) can enhance the mechanical strength of insect cuticles (^15^ and references therein).

In beetles, the order representing almost 25% of described animal species (~ 400,000), the most strikingly coloured parts are the elytra^2,16^. Elytral colours serve a variety of adaptive functions, e.g. camouflage, mimicry, antipredator cues, species and sex recognition, and thermal balance^2,16–18^. The colours may be due to chemical pigments (known as chemochromes), but they can also be structural (i.e. specific physical structures; reviewed in^2,3^). The latter are caused by the diffraction or multilayer interference of light in micro-anatomical features and the variable thickness of the different layers in the body integument^19–21^. In some beetles, structural colours are reversible as a result of moisture absorption: this alters the thickness of the transparent films, thereby changing the reflectance spectrum (reviewed in^2,18^).

Beetle elytra consist of three major layers—the epi-, exo-, and endocuticles; but the coloured patches/patterns on the elytra are due primarily to the pigmentation of the two outermost layers^2,16,19^. The quantitative data obtained in some rare, earlier studies using scanning electron microscopy (SEM) show that each of these three layers can itself vary in elemental composition, a property that can be explained by the different associations with or origins of the individual elements in various subcellular structures and macromolecules^22,23^. Another interesting, recent study on single insects representing 30 species from 13 families aimed to map the spatial variation of ten trace elements in differently-coloured cuticular regions^24^. This study showed that trace element concentrations in cuticular regions of similar colour in different taxa resembled each other. The interspecific variations in dark, melanin-rich regions of the cuticle was explained by differences in concentrations of K, Zn and to a lesser extent, Ca, while S, Mn and Fe were responsible for variation in the red regions of the cuticle^24^. Owing to the small sample size, however, those findings say nothing about the variation in elemental composition within a population of a single species, which could be attributed to variability in colour intensity, diet/resourcesor sexual differences.

**Figure 1:**
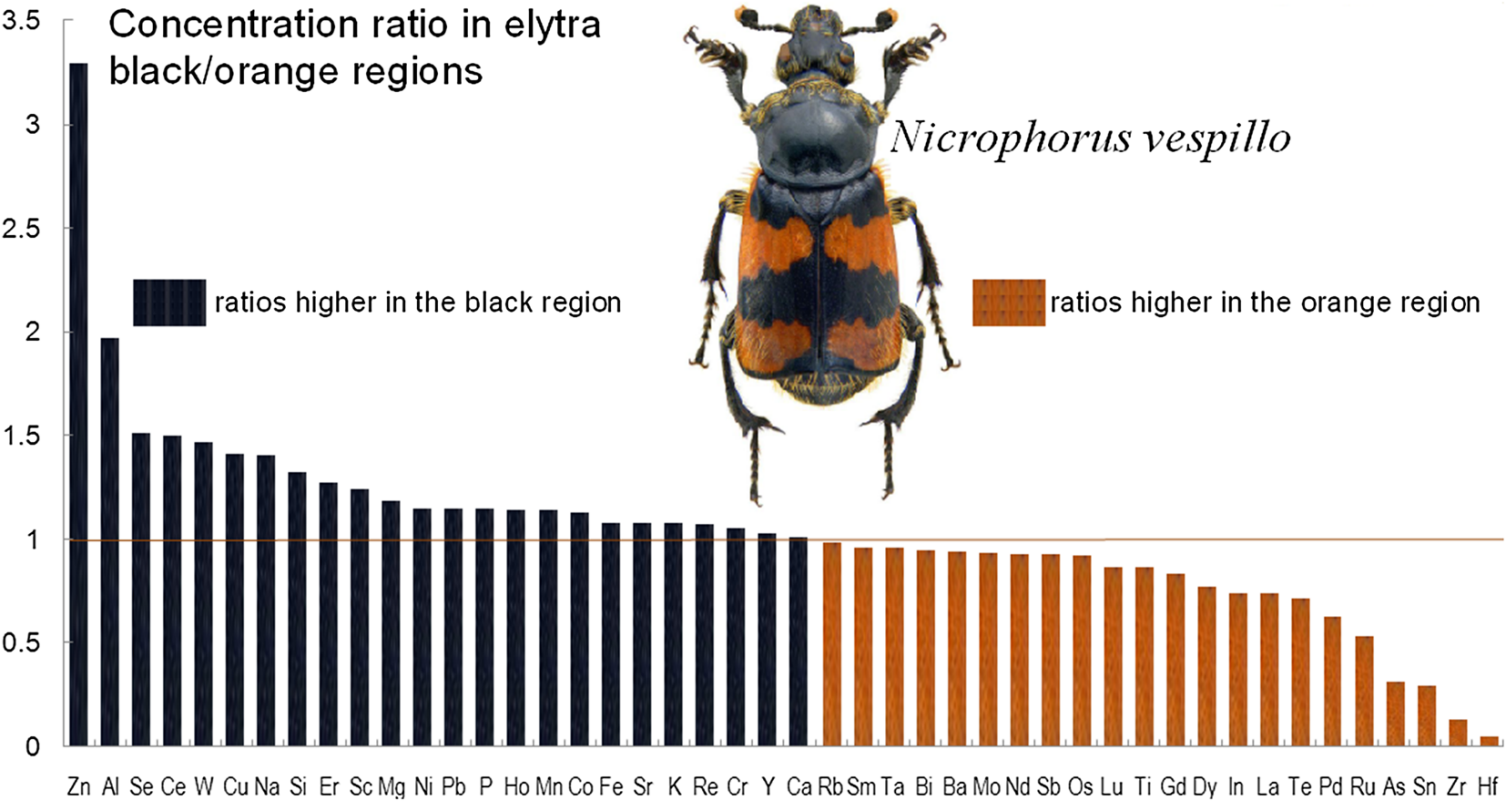
Ratios (*y*-axis) of concentrations of chemical elements measured in two adjacent regions of an insect cuticle with a contrasting colour pattern, i.e. the black and orange elytral regions of the aposematic burying beetle *Nicrophorus vespillo*. The elements are shown in decreasing order of ratios: bars with values > 1 (orange line) represent elemental concentrations higher in the black region, while bars with values < 1 represent concentrations higher in the orange region (for elemental concentrations and sample sizes, see Table 1). The photo of the species is by Udo Schmidt from Germany: *Nicrophorus vespillo* (Linné, 1758), male, uploaded by Magnus Manske, CC BY-SA 2.0, taken from https://commons.wikimedia.org/w/index.php?curid=22950523. This file is licensed under the Creative Commons Attribution-Share Alike 2.0 Generic license, which allow freely to share (to copy, distribute and transmit the work), and to remix (to adapt the work). *Note*: The upper surfaces of the elytra are bi-coloured black and orange, whereas the under surfaces (those adhering to the body) are partly orange (see also^18^); rough measurements of the thickness of the black and orange layers in the elytra of *N. vespillo* show that, depending on their location on the elytra, the thickness of the black layer ranged from *c.* 0.03 to 0.1 mm, on average 30–50% of the entire elytral thickness, whereas the orange layer was between 0.07 and 0.2 mm thick.

In the present study, we used inductively-coupled plasma optical emission spectrometry (ICP-OES) to determine ionomic profiles and their variability in microsamples of two adjacent regions of an insect exoskeleton with a contrasting warning colour pattern: specifically, the black and orange regions isolated from elytra of the aposematic burying beetle *Nicrophorus vespillo* (Coleoptera, Silphidae). The study species is a good model for assessing colour-assisted differences in elytral elemental composition because, like most other burying beetles, *N. vespillo* has two bright orange stripes on its elytra^18,25^ that stand out against the black ground colour (Fig. 1). The orange-black elytral markings of burying beetles function as an aposematic warning display^25^. Thus, in the broader ecological sense, we ask here whether there exists a functional correspondence between the aposematic colours of insect exoskeletons and their ionomic profiles, and whether differences in the content of some of the chemical elements can explain the effect of the aposematic colouration in beetles with a warning colour pattern. Generally speaking, organisms with warning colours are often unpalatable or even poisonous—chemical secretions produce strong olfactory signals to their potential predators^25,26^. It is unclear, however, whether the level of toxicity of these secretions (aposematic effect) can be predicted from high levels of certain elements (metals) in the body integument. By analogy, it was found in the Asian ladybird *Harmonia axyridis* that individuals with proportionally more red (due to carotenoid pigments) on the elytra were more alkaloid-rich; elytra colour patterns thus have the potential to reveal information about stronger chemical defences^27^.

## Methods

Specimens of *N. vespillo* were obtained from pheromone traps used in commercial forests to catch the European spruce bark beetle *Ips typographus*. The traps were deployed in small forest complexes (52° 05′ 23**ʺ** N 16° 24′ 01ʺ E) in the Ziemin Forest District (Kościan Forest Inspectorate; SW Poland), and the use of such traps in forests by forestry services does not require special permits. All the material was obtained in June–August 2017; and all the specimens of *N. vespillo* used in the study, were provided to us by the forest services. The presence of *N. vespillo* in such traps is of a secondary nature: they had been lured there by the odour of wet insect carcasses, decomposing as a result of the poor drainage of rainwater from the traps^28^.

The orange and black elytral fragments were isolated under a binocular microscope (× 10) using a microscalpel. A single sample consisted of the relevant elytral fragments from six beetles. A total of 240 specimens of *N. vespillo* were used in the study: this yielded 39 samples with orange fragments and 39 samples with black fragments (see Supplementary Information file, Table S1). Samples of orange (marked "A") and black (marked "B") fragments were placed in pairs in plastic bags and numbered.

### Chemical analysis

#### Instruments

An inductively-coupled plasma optical emission spectrometer (Agilent 5110, Agilent, USA) was used to determine the elemental composition of the samples^29^. The simultaneously axial and radial view of the plasma was enabled by synchronous vertical dual view (SVDV). The standard conditions were applied: Radio Frequency (RF) power 1.2 kW, nebulizer gas flow 0.7 L min^−1^, auxiliary gas flow 1.0 L min^−1^, plasma gas flow 12.0 L min^−1^, viewing height for radial plasma observation 8 mm, detector CCD (Charge Coupled Device) temperature  − 40 °C, signal accusation time 5 s for 3 replicates. A Mars 5 (CEM, Matthews, USA) microw ave sample preparation system was used for sample digestion^29,30^.

#### Procedure

Accurately weighed (range; ± SE) 0.0027–0.0091 g (± 0.00013 g) dry samples were digested in 2 mL concentrated nitric acid in closed Teflon containers in a Mars 6 (CEM, USA) microwave sample preparation system. After digestion, the samples were diluted with water to a final volume of 6.0 mL.

#### Analytical method validation

The detection limits were estimated at the level of 0.01 mg kg^−1^ dry weight (ppm. d.w.) for all the elements determined (as 3-sigma criteria). The uncertainty of the overall analytical procedure (including sample preparation) was 20%. Traceability was checked using reference materials: CRM S-1—loess soil; CRM NCSDC (73,349)—bush branches and leaves; CRM 2709—soil; CRM 405 and CRM 667—estuarine sediments. The recovery (80–120%) was acceptable for most of the elements determined. The recovery of the uncertified elements was defined by the standard addition method^30^.

### Statistical treatment

Initially, we assessed the overall frequency of element detectability (i.e. measurement above the detection limit) in the orange *versus* black elytral regions (*n* = 39 in each case), using the *t*-test for paired comparisons and its non-parametric equivalent, the Wilcoxon signed rank test, which takes into account the small number of paired observations for many elements.

Owing to the very considerable variance in the concentrations of some elements and the large number of non-detects (excluded from our major quantitative analysis) among the measurements (see “Results”), most results are presented in a descriptive fashion. For the same reasons, we employed two different approaches to data analysis when calculating average elemental concentrations and assessing potential differences between the black and orange regions of the elytra: (i) collectively across all the beetles examined, and (ii) measured simultaneously as pseudo-pairs per group of six individual beetles.

First, we examined the overall differences in concentrationsof individual elements, as well as the total concentration (∑) of all elements and the sum of different REEs between the black and orange elytral regions across all the available data points (Table 1; Supplementary Information file, Table S1). We used one-way ANOVA to test this, checking the variables (elemental concentrations) for normality (Kolmogorov–Smirnov test) for all ANOVAs. When necessary, data *log*-transformations were carried out to meet the parametric assumption prior to analysis. In principle, however, this treatment had no effect on the final ANOVA results. Moreover, because *log*-transformation did not improve the distribution of some variables, we repeated the between-samples comparison using the non-parametric Mann–Whitney test.

**Table 1:** Average (± SE) concentrations (ppm d.w.) of chemical elements measured by ICP-OES in two adjacent regions of an insect cuticle with a contrasting colour pattern, namely, the black and orange regions of the elytra of the aposematic burying beetle *Nicrophorus vespillo* (Coleoptera: Silphidae; see Fig. 1).

Element^†^	Orange region (*n* = 39)	Black region (*n* = 39)	Element	Orange region (*n* = 39)	Black region (*n* = 39)
Al	**0.96 ± 0.10 (9)**	**1.89 ± 0.29 (12)**	Ni	0.095 ± 0.023 (15)	0.109 ± 0.029 (14)
	[0.34–1.29]	[0.35–4.31]		[0.017–0.332]	[0.024–0.416]
As	1.21 ± 0.73 (8)	0.381 ± 0.08 (8)	Os	0.026 ± 0.004 (12)	0.024 ± 0.004 (12)
	[0.12–6.25]	[0.07–0.63]		[0.011–0.057]	[0.011–0.061]
Ba	2.66 ± 0.57 (39)	2.51 ± 0.47 (39)	P	11.10 ± 1.40 (39)	12.71 ± 1.58 (39)
	[0.29–11.85]	[0.33–11.96]		[0.722–41.20 ]	[1.40–49.46]
Bi	0.095 ± 0.024 (9)	0.090 ± 0.040 (5)	Pb	0.334 ± 0.034 (38)	0.383 ± 0.033 (39)
	[0.018–0.236]	[0.024–0.241]		[0.076–1.09]	[0.026–1.13]
Ca	41.86 ± 7.13 (37)	42.33 ± 6.86 (39)	Pd	0.016 ± 0.003 (5)	0.010 (1)
	[0.103–148.4]	[0.712–137.5]		[0.010–0.024]	–
Cd	BDL	0.014 ± 0.001 (3)	Pr	0.019 ± 0.002 (17)	0.018 ± 0.001 (16)
	–	[0.012–0.015]		[0.011–0.041]	[0.011–0.032]
Ce	0.018 ± 0.004 (3)	0.027 ± 0.012 (2)	Rb	4.01 ± 0.12 (39)	3.96 ± 0.13 (39)
	[0.011–0.025]	[0.015–0.039]		[2.70–5.46]	[2.53–5.28]
Co	0.023 ± 0.002 (16)	0.026 ± 0.003 (18)	Re	0.056 ± 0.013 (8)	0.060 ± 0.011 (14)
	[0.011–0.043]	[0.011–0.056]		[0.013–0.107]	[0.010–0.142]
Cr	0.019 ± 0.002 (8)	0.020 ± 0.003 (7)	Rh	0.019 ± 0.004 (2)	0.019 (1)
	[0.013–0.027]	[0.0101–0.037]		[0.015–0.023]	–
Cu	**1.26 ± 0.13 (39)**	**1.78 ± 0.20 (39)**	Ru	0.028 ± 0.011 (3)	0.015 ± 0.001 (3)
	[0.386–3.70]	[0.441–5.20]		[0.011–0.049]	[0.014–0.017]
Dy	0.026 ± 0.005 (7)	0.020 ± 0.005 (10)	Sb	5.49 ± 0.86 (39)	5.08 ± 0.30 (39)
	[0.010–0.048]	[0.011–0.054]		[0.819–35.47]	[1.40–9.07]
Er	0.011 ± 0.001 (2)	0.014 (1)	Sc	0.025 ± 0.005 (8)	0.031 ± 0.009 (6)
	[0.010–0.012]	–		[0.010–0.060]	[0.010–0.066]
Fe	2.21 ± 0.26 (39)	2.39 ± 0.24 (39)	Se	0.111 ± 0.019 (11)	0.168 ± 0.035 (12)
	[0.637–9.79]	[0.818–8.15]		[0.039–0.259]	[0.025–0.387]
Gd	0.018 ± 0.003 (5)	0.015 ± 0.002 (8)	Si	4.16 (1)	5.50 (1)
	[0.010–0.029]	[0.010–0.023]		–	–
Ge	BDL	0.031 ± 0.005 (3)	Sm	0.026 ± 0.003 (8)	0.025 ± 0.003 (8)
	–	[0.021–0.037]		[0.012–0.034]	[0.010–0.036]
Hf	1.352 ± 1.293 (4)	0.070 ± 0.038 (4)	Sn	0.303 ± 0.196 (8)	0.090 ± 0.018 (16)
	[0.014–5.23]	[0.025–0.185]		[0.021–1.66]	[0.013–0.256]
Ho	0.014 ± 0.001 (6)	0.016 ± 0.004 (3)	Sr	0.129 ± 0.021 (26)	0.139 ± 0.019 (25)
	[0.011–0.017]	[0.011–0.024]		[0.013–0.357]	[0.012–0.281]
In	0.198 ± 0.041 (10)	0.147 ± 0.025 (5)	Ta	0.025 ± 0.005 (10)	0.024 ± 0.005 (7)
	[0.030–0.387]	[0.108–0.245]		[0.010–0.066]	[0.013–0.045]
K	22.13 ± 1.45 (39)	23.83 ± 1.93 (39)	Tb	0.0196 ± 0.002 (26)	0.0203 ± 0.002 (20)
	[10.24–52.01]	[10.76–73.39]		[0.010–0.045]	[0.012–0.039]
La	0.042 ± 0.006 (18)	0.031 ± 0.006 (17)	Te	0.227 ± 0.032 (25)	0.163 ± 0.025 (22)
	[0.010–0.101]	[0.010–0.097]		[0.022–0.853 ]	[0.018–0.481]
Lu	0.015 ± 0.001 (7)	0.013 ± 0.001 (8)	Ti	0.030 ± 0.005 (35)	0.026 ± 0.003 (36)
	[0.011–0.022]	[0.010–0.019]		[0.010–0.174]	[0.011–0.102]
Mg	4.96 ± 0.82 (39)	5.88 ± 0.78 (39)	V	BDL	0.027 (1)
	[0.182–19.65]	[0.460–16.72]		–	–
Mn†	**0.408 ± 0.051 (39)**	**0.466 ± 0.038 (39)**	W^†^	**0.064 ± 0.013 (19)**	**0.094 ± 0.012 (26)**
	[0.104–1.55]	[0.184–1.30]		[0.011–0.271]	[0.017–0.271]
Mo	0.032 ± 0.001 (5)	0.030 ± 0.006 (7)	Y	0.037 ± 0.007 (16)	0.038 ± 0.006 (11)
	[0.029–0.035]	[0.014–0.061]		[0.013–0.117]	[0.010–0.075]
Na	7.22 ± 1.18 (38)	10.15 ± 1.51 (37)	Zn	0.408 ± 0.025 (39)	0.409 ± 0.026 (39)
	[0.809–28.07]	[1.86–32.49]		[0.134–0.823]	[0.131–0.737]
Nb	0.168 (1)	BDL	Zr	0.576 ± 0.469 (25)	0.075 ± 0.028 (25)
	–	–		[0.010–11.80]	[0.020–0.725]
Nd	0.042 ± 0.005 (37)	0.039 ± 0.005 (33)	REEs‡	0.120 ± 0.017(39)	0.099 ± 0.014 (39)
	[0.010–0.143]	[0.011–0.114]		[0.010–0.479]	[0.021–0.355]

The number of samples exceeding the detection limits and the ranges of concentrations are given in round and square brackets, respectively. BDL—all samples below the detection limit (0.01 ppm d.w.). Significant differences (*P* ≤ 0.05) obtained in ANOVA or with the Mann–Whitney test are in bold.

The concentrations of Ag, B, Be, Eu, Ga, Hg, Li, Tl, Tm and Yb in all the samples were BDL.

^†^The statistically significant differences for Mn and W were obtained using the Mann–Whitney test: *U* = 519 and 156, *P* = 0.015 and 0.037.

^‡^The sum of REEs (rare earth elements) includes the concentrations of Ce, Dy, Er, Gd, Ho, La, Lu, Nd, Pr, Sc, Sm, Tb and Y.

Second, to reduce the large overall variance in elemental concentrations within the entire population of *N. vespillo* with the aim of achieving more consistent results in the context of individual variation, the data on elemental concentrations were arranged in the form of pseudo-pairs, i.e. corresponding measurements of orange ("A") and black ("B") elytral samples derived from six beetles (Table 2). We used the *t*-test for dependent samples to test the differences for these paired measurements. Owing to the large number of non-detects for some chemical elements, fewer samples (i.e. simultaneous paired observations for orange and black elytral samples) were used in the *t*-test analysis than for the full data set presented in Table 1.

**Table 2:** Average (± SE) concentrations (ppm d.w.) of the chemical elements measured simultaneously in pseudo-pairs in two adjacent regions of an insect cuticle with a contrasting colour pattern, namely, the black and orange regions of the elytra of the aposematic burying beetle *Nicrophorus vespillo* (Coleoptera: Silphidae).

Element (number of pairs)	Orange region	Black region	Element (number of pairs)	Orange region	Black region
Al (7)	1.049** ± **0.087	1.638 ± 0.330	Os (4)	0.027 ± 0.003	0.025** ± **0.012
As (3)	0.497 ± 0.261	0.366** ± **0.142	P (38)	11.31** ± **1.42	12.76 ± 1.62
Ba (38)	2.714 ± 0.581	2.555** ± **0.483	Pb (37)	0.337** ± **0.035	0.394 ± 0.034
Bi (2)	0.135 ± 0.044	0.043** ± **0.018	Pr (8)	0.0164 ± 0.002	0.0155** ± **0.001
Ca (36)	42.99** ± **7.24	45.48 ± 7.18	Rb (38)	4.041 ± 0.123	3.972** ± **0.135
Co (11)	0.024** ± **0.003	0.026 ± 0.005	Re (3)	0.081 ± 0.019	0.020** ± **0.009
Cr (2)	0.021 ± 0.002	0.014** ± **0.004	Rh (1)	0.015	0.019
Cu (38)	**1.279 ± 0.127**	**1.787 ± 0.207**	Sb (38)	5.58 ± 0.88	5.11** ± **0.31
Dy (2)	0.027 ± 0.001	0.018** ± **0.005	Sc (3)	0.022 ± 0.005	0.019** ± **0.007
Fe (38)	2.24** ± **0.27	2.43 ± 0.24	Se (4)	**0.074 ± 0.025**	**0.146 ± 0.032**
Gd (2)	0.023 ± 0.006	0.018** ± **0.004	Sm (2)	0.021** ± **0.009	0.028 ± 0.001
Hf (1)	5.231	0.185	Sn (3)	0.132 ± 0.055	0.072** ± **0.047
Ho (1)	0.012	0.024	Sr (22)	0.147** ± **0.023	0.148 ± 0.021
In (1)	0.162	0.108	Ta (3)	0.018** ± **0.005	0.026 ± 0.008
K (38)	22.32** ± **1.48	23.98 ± 1.98	Tb (14)	0.024 ± 0.003	0.023** ± **0.002
La (9)	0.043 ± 0.011	0.038** ± **0.007	Te (17)	0.217 ± 0.045	0.161** ± **0.030
Lu (3)	0.014 ± 0.002	0.013** ± **0.002	Ti (31)	0.032 ± 0.006	0.027** ± **0.004
Mg (38)	5.087** ± **0.828	5.999 ± 0.788	W (13)	0.068** ± **0.019	0.110 ± 0.022
Mn (38)	0.413** ± **0.052	0.470 ± 0.039	Y (8)	0.041 ± 0.012	0.040** ± **0.007
Mo (1)	0.032	0.027	Zn (38)	0.416 ± 0.026	0.413** ± **0.026
Na (35)	**7.26 ± 1.27**	**10.60 ± 1.56**	Zr (18)	0.774 ± 0.650	0.088** ± **0.038
Nd (31)	0.046 ± 0.005	0.040** ± **0.005	∑ all elements (38)	105.32** ± **7.85	114.49 ± 8.13
Ni (6)	0.075** ± **0.012	0.144 ± 0.057	REEs (38)	0.122 ± 0.017	0.102** ± **0.014

Significant differences (*P* ≤ 0.05) for paired comparisons (*t*-test for dependent samples) are in bold; the higher concentration is underlined.

(1) Concentrations of Cd, Ce, Er, Eu, Ga, Ge, Hg, Li, Nb, Pd, Ru, Si, Tl, Tm, V and Yb were not measured simultaneously for any pairs.

(2) The variances (σ^2^ ≥ 0.011) in concentrations of the following elements measured in the orange and black elytral regions were relatively high: Al (0.05 l; 0.76), As (0.20; 0.06), Ba (13; 8.9), Ca (1887; 1858), Cu (0.61; 1.63), Fe (2.7; 2.2), K (83; 148), Mg (26; 24), Mn (0.10; 0.06), Na (57; 85), Ni (0.001; 0.019), P (77; 100), Pb (0.044; 0.042), Rb (0.57; 0.70), Sb (29; 3.7), Te (0.03; 0.02), Zn (0.026; 0.025), Zr (7.6; 0.03), ∑ all elements (2343; 2512) and REEs (0.011; 0.008); the variances for all the other elements were always ≤ 0.011.

The statistical analyses were performed using Statistica ver. 12.5^31^ and Excel software. A probability of *P* < 0.05 was considered statistically significant.

### Ethics declarations

We did not employ invasive techniques for this study.

## Results

ICP-OES analysis of 78 fragments of orange and black elytral regions from *N. vespillo* (Fig. 1) revealed 53 elements (ranging in atomic weight from Na to Bi) present above the detection limit (Table 1). The frequency of detectability of individual elements varied strongly, however, and only ten elements (Ba, Cu, Fe, K, Mg, Mn, P, Rb, Sb and Zn) occurred in concentrations exceeding the detection limit in each sample. The number of non-detects for the remaining elements varied greatly from 1 (Pb) to as many as 77 (Nb and V) (Table 1). We found no statistical differences in the frequency of detectabilityof the 53 elements in the orange *versus* black elytral regions (*n* = 39 in each case) when using either the *t*-test or the Wilcoxon test for paired comparisons (*P* ≥ 0.771).

The overall concentration (∑ concentrations) of all 53 elements (Table 1) was low, being merely 112.8 (95% CI 96.4; 129.2) ppm d.w. and 103.5 (95% CI 87.2; 119.4) ppm d.w. in the black and orange elytral regions, respectively. Correspondingly, the total concentration of all the elements in the orange regions was 9% lower than in the black ones. However, this difference was not statistically significant (one-way ANOVA, F_1,76_ = 0.683, *P* = 0.411), presumably because of the mostly extremely large variance in overall concentration (σ^2^ = 2558 and 2417 in the black and orange regions, respectively).

The rare earth elements exhibited the opposite distribution. The sum of REEs included the concentrations of 13 elements (Ce, Dy, Er, Gd, Ho, La, Lu, Nd, Pr, Sc, Sm, Tb and Y) and was 17% lower in the black regions of the elytra than in the adjacent orange ones (Table 1). The variance in the sum of REE concentrations measured in the black and orange regions was negligible (σ^2^ = 0.08 and 0.011, respectively), and the difference between these two samples did not approach statistical significance (ANOVA, F_1,76_ = 0.923, *P* = 0.340).

**Figure 2:**
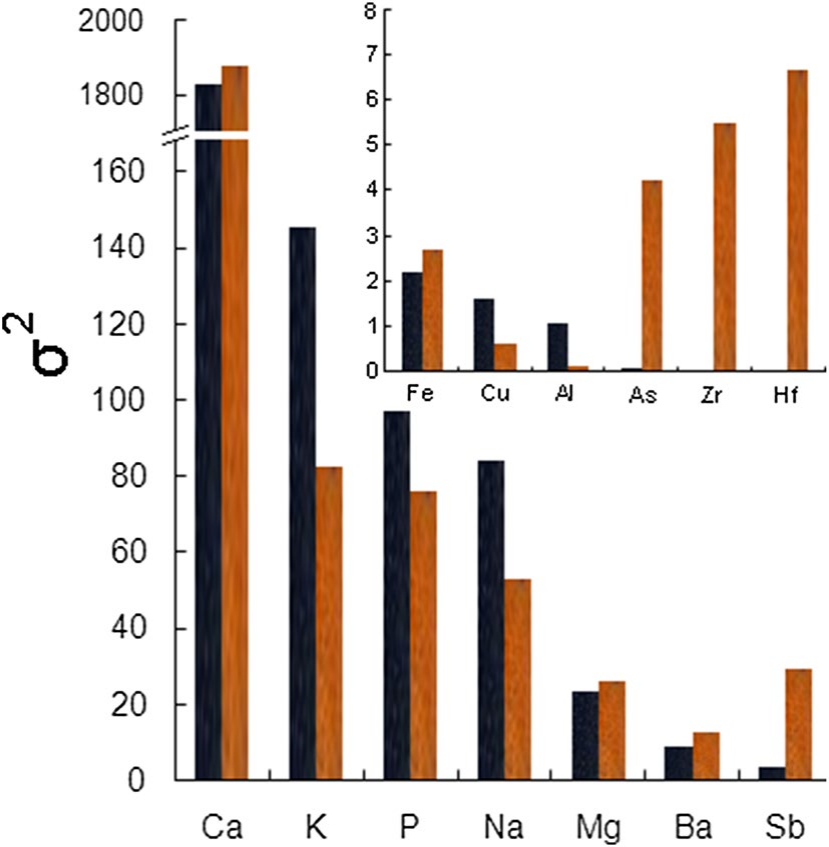
Thirteen elements measured in the black and orange regions of the elytra with variance (σ^2^) of concentrations > 1; elements shown in order of decreasing variance. The small panel depicts elements with relatively lower variances in their concentrations: the respective variances for As, Zr and Hf measured in the black regions were 0.052, 0.019 and 0.006, while that for Al measured in the orange regions was 0.09. The variance in concentration of all the other elements listed in Table 1 in both these elytral regions was always ≤ 0.69.

Further analysis of the variance in concentrations of individual elements showed that 14 elements displayed variances > 1 (Fig. 2). Specifically, we found an enormous variance in Ca concentrations (σ^2^ > 1800); at the same time, this was the most abundant element, making up an average of 30.2% (95% CI 25.0%; 35.5%; *n* = 76) of the sum of all elemental concentrations per sample. Concentrations of 12 other elements (K, P, Na, Mg, Ba, Sb, Fe, Cu, Al, As, Zr and Hf) exhibited variances of up to 145, although this was also dependent on the colour of the elytra sample, e.g. the variances in K, P and Na concentrations were much higher in the black region (Fig. 2). The variance in concentration of all the other elements listed in Table 1 was negligible, always ≤ 0.69.

To visualize the variation in the distribution of individual elements, we calculated the ratios of their concentrations measured in the black and orange elytral regions in all the samples (Fig. 1). For this purpose, we used the concentrations of 48 elements, for which sample sizes allowed this calculation (see Table 1). Interestingly, we found that the concentrations of half (24) of these elements were higher in the black elytral region (Fig. 1), ranging from 1.011 (Ca) to 1.969 (Al). In contrast, the concentrations of the other 24 elements were higher in the orange regions of the elytra than in the black ones, the difference ranging from 0.052 (Hf) to 0.998 (Zn).

More detailed analysis for individual elements, based on a rigorous statistical treatment aimed at assessing the differences between the black and orange regions of the elytra, showed that the concentrations of four elements varied significantly between these two samples—Al (ANOVA, F_1,19_ = 6.94, *P* = 0.016) and Cu (ANOVA, F_1,76_ = 4.79, *P* = 0.032)—and in the non-parametric analysis for Mn and W. Specifically, the concentrations of these four elements in the black elytral regions were respectively higher by 97%, 41%, 14% and 47% than in the orange regions (see Table 1 for elemental concentrations, and the results of the Mann–Whitney test for Mn and W).

Lastly, the analysis using the *t*-test for dependent samples based on data on elemental concentrations set up as pseudo-pairs (i.e. corresponding measurements from six different beetles), presented in Table 2, shows that concentrations of Cu (t_37_ = − 2.67, *P* = 0.011), Na (t_34_ = − 2.18, *P* = 0.036) and Se (t_3_ = − 5.03, *P* = 0.015) were significantly higher in the black regions than in the orange regions of the elytra by 40%, 46% and 97%, respectively. Furthermore, another marginally significant difference was displayed by the Al concentration (t_6_ = − 2.11, *P* = 0.079), being 56% higher in the black regions of the elytra (Table 2).

## Discussion

In this paper we provide novel and basic quantitative information on the concentrations of tens of chemical elements occurring within a specific animal tissue, an insect exoskeleton, and confirm the potential usefulness of ICP-OES as an effective tool for determining the ionomic profiles of microsamples of highly sclerotized insect tissues. Based on our estimated concentration ratios of elements measured in the black and orange elytral regions of *N. vespillo*, our results suggest that the distributions of all the elements combined, rare earth elements and nearly every individual element are unequal between these two differently coloured parts of the elytra, although the trend of these associations varies between elements. In a broader sense, our study appears to confirm the idea that dark (melanin-rich) regions can act as sinks/reservoirs for certain trace elements/metals^5^. This was the case for *inter alia* Al, Se, Ce, W, Cu, Na, Si, Er, Sc, Mg, Ni and Pb, whereas a group of other elements (Lu, In, La, Te, Pd, Ru, As, Sn, Zr and Hf) tended to be concentrated in the orange elytral regions.

It should be emphasized, however, that rigorous statistical treatment yielded much less ambiguous results and that concentrations of only a minority of elements varied significantly between the black and orange elytral parts. Specifically, depending on the type of data analysis approach (across all beetles, and measured simultaneously as pseudo-pairs), we found that the black elytral regions were apparently characterized by higher concentrations of Al, Cu, Mn and W (demonstrated by one or both of these approaches) and Na and Se (pseudo-pairs). Basically, close inspection showed that the concentrations of these six elements obtained with both these approaches (cf. Tables 1 and 2) were broadly consistent, confirming their apparently higher levels in the black elytral region. This is also clearly evident from Fig. 1, where all these elements are placed on the left-hand side of that chart. In addition, we found that the between-sample differences for almost all the other elements listed in Tables 1 and 2 showed consistent trends. Thus, in spite of some minor inconsistencies (e.g. for Tb or Y), these two approaches are relevant to investigations of colour-assisted variation in elemental composition in our sample of beetle elytra. It is hard to state unequivocally, however, that the above-mentioned metals (or others, like REEs) are responsible for the differences in colouration between the black and orange elytral regions of *N. vespillo*. More importantly, since our samples are cross-sections through all the elytral layers, establishing such a relationship would require the examination of uniformly coloured layers of the beetle elytra.

Another important aspect of our investigation was to describe the magnitude of the variability in the concentrations of different elements. In particular, there was very considerable variance in the concentrations of essential elements (Ca, K, P, Na, Mg and Ba) measured in both the black and orange elytral regions (see Fig. 2). Such a large variability is extremely surprising, and at present we are unable to pinpoint the exact reasons for this. We may speculate that such a high variance in essential element concentrations could in part be attributed to individual differences, e.g. those resulting from the consumption of animal carcasses of different quality/chemical composition, or from sexual differences, especially that female burying beetles spend much more time at a carcass caring for their offspring than males^25,32^. Furthermore, the intensity of colouration of both the orange and black elytral markings of burying beetles is variable (as found in a related species of burying beetle,*N. vespilloides*^25^), which may be responsible for the different content of pigments and/or elements. Moreover, as a form of chemical (antipredator) defence, both male and female burying beetles secrete anal exudates, with females producing more of this fluid^25^; to some degree, this could also affect the entire metal pool in the body. Consequently, the depletion of essential elements (or their intensive passage/trafficking) also seems to be more likely in females. Lastly, interferences between elements (in particular Ca) and the resulting lowering of measurement quality are also possible (e.g.^33^). On the other hand, the variances in concentrations of other elements, including REEs, are low (σ^2^ ≤ 0.69), which in combination with their low concentrations indicate either stringent physiological control or a poor ability to bind with the exoskeleton.

Importantly, because the concentrations of chemical elements in different coloured regions of the insect integument were not measured in previous studies, we can only compare our data with those earlier studies in a broader ecotoxicological context, i.e. at the level of the whole body and the chitinous integument. Consequently, inspection of the concentrationswe obtained, which are basically at normal physiological levels given the whole-body concentrations of the vast majority of elements (cf.^10,13,34^), reveals high levels of As and Sb. Another recent study has shown that large amounts of As (up to 254.9 As ppm d.w.) bind with beetle elytra^12^. At the same time, whole-body concentrations of Sb in different invertebrate taxa were generally much lower (up to 3.26 Sb ppm d.w.^35^) than in our study (up to 35.47 Sb ppm d.w.; Table 1). Nonetheless, there are data showing much higher Sb concentrations in organisms from contaminated mine sites (up to 66.4 Sb ppm d.w.^36^). It remains an open question, however, whether the high concentrations of these metalloids can be related to the insecticides used in the pheromone traps from which we collected the analytical material.

## Conclusions and research perspectives

Two major conclusions with obvious research perspectives can be drawn from our study.

Firstly, in a broader perspective, beetle elytra or insect exoskeletons are readily available, e.g. from remains following a predation event, faeces, pellets, guano deposits, roadkill, museum collections or even palaeobiological material, so further studies employing such analytical material in the biomonitoring of environmental quality are both feasible highly desirable. Such studies permit an effective assessment of changes in environmental quality, with respect to both the historical context^37^ and current land use^12^.

Secondly, at the organism level, various analytical techniques are used to determine the content of chemical elements in biological samples: atomic absorption spectrometry (FAAS and/or GFAAS), inductively coupled plasma with detection of optical emission spectrometry (ICP-OES), and mass spectrometry (ICP-MS). All of these techniques are equally relevant and useful. From the methodological point of view, it should borne in mind that in our study, ICP-OES measured the concentrations of a large number of elements, but at a relatively high detection limit (> 0.01 ppm; see “Methods”). Some trace elements, however, in particular those associated with pigments present in thin layer(s) of elytra or specific subcellular structures and/or cuticular macromolecules (e.g. lipids/wax, proteins; reviewed in^38^), most likely occur in different coloured parts of beetle elytra (or various parts of an insect’s parti-coloured integument) in much lower concentrations. The one basic issue relating to our methodology is that the drawing of logical inferences regarding the source of differences in the elemental composition of insect exoskeletons or beetle elytra is that our sampling covered the entire thickness of the chitninous integument. This clearly implies the need for future studies employing many more throughput methods of chemical analysis for determining such low contents of chemical elements in individual insect micro-samples. We highlight the fact that progress in understanding the basic relationship between insect colouration and variation in elemental composition requires the micro-sampling of the homogeneous layers of exoskeletons. Consequently, although the ecological significance of our study seems to be weak, given the absence of an argument that there is a functional correspondence between the colour of elytra and their variability in the ionomic profile, we strongly encourage further exploration of the basic question on metal partitioning between differently coloured parts of sclerotized insect integuments in order to account for individual differences, e.g., sex, body size and colour variation.

## Supplementary Information

Supplementary Information

## Data Availability

All the data analysed during this study are included in this published article (and its Supplementary Information file). Table S1: Concentrations (ppm d.w.) of chemical elements measured by ICP-OES in the black and orange regions of the elytra of the burying beetle *Nicrophorus vespillo*.
